# High-risk pregnancy in India: Prevalence and contributing risk factors – a national survey-based analysis

**DOI:** 10.7189/jogh.13.04116

**Published:** 2023-09-15

**Authors:** Periyasamy Kuppusamy, Ranjan Kumar Prusty, Deepali Prakash Kale

**Affiliations:** 1 Clinical Research Laboratory, ICMR-National Institute for Research in Reproductive and Child Health, Mumbai, India; 2 Department of Biostatistics, ICMR-National Institute for Research in Reproductive and Child Health, Mumbai, India; 3 Department of Obstetrics and Gynaecology, Nowrosjee Wadia Maternity Hospital, Mumbai, India

## Abstract

**Background:**

High-risk pregnancies (HRP) place women and their offspring at the highest risk for morbidity and mortality. Maternal and medical risks increase pregnancy risk and complications during pregnancy and childbirth. Here, we reported the current prevalence of high-risk pregnancies among Indian women, which is defined through various factors such as maternal, lifestyle, medical, current health risk and adverse birth outcomes.

**Methods:**

This is a cross-sectional study based on secondary data from India’s National Family Health Survey-5 (NFHS-5). A total of 23 853 currently pregnant women were considered for analysis after considering the inclusion and exclusion criteria. The prevalence and contributing factors of high-risk pregnancies were estimated using descriptive statistics and logistic regression, respectively.

**Results:**

The prevalence of high-risk pregnancies among Indian women was 49.4%, with 33% of women having a single high-risk, and 16.4% having multiple high-risk pregnancies. Notably, pregnant women from Meghalaya and Manipur states had 67.8% and 66.7% with one or more high-risk factors, respectively. About 31.1% of women had short birth spacing, and 19.5% of women had adverse birth outcomes during the last birth. Logistic regression analysis showed that women with no education (adjusted odds ratio (AOR) = 2.02; 95% confidence interval (CI) = 1.84-2.22) and the poorest wealth quintile (AOR = 1.33; 95% CI = 1.04-1.29) had significantly higher odds of having HRP than those with higher education and the highest wealth quintile, respectively.

**Conclusions:**

Nearly half of all pregnancies in India have one or more high-risk factors, which is a matter of concern, and the risks were higher among the vulnerable population such as no educated, poorest groups etc. The leading high-risk factors such as short-birth spacing, adverse birth outcomes, and caesarean deliveries should be addressed through the health policy and programmes.

Globally, maternal deaths due to complications from pregnancy or childbirth were estimated at 211 deaths per 100 000 live births in 2017 [1]. About 1.3 million maternal deaths were estimated among Indian women in the last two decades [2] accounting for 12% of global maternal deaths [1]. The majority of maternal deaths were due to direct medical causes [3]. According to the Sample Registration System, the maternal mortality ratio (MMR) has declined from 113 deaths per 100 000 live births between 2016 and 2018 to 103 deaths per 100 000 live births between 2017 and 2019, and the majority of maternal deaths occurred in the age range of 20-29 years [4]. Poor maternal health indicators are directly associated with pregnancy-related morbidities and mortality. Factors such as severe bleeding (47.0%), pregnancy-related complications (19.6%), infection or sepsis (12.0%), pregnancy-induced hypertensive disorders (7.0%), and abortions (5.0%) were the leading causes of maternal deaths in India [2]. With high maternal mortality rates in India, a strategic programme on Reproductive, Maternal, New-born, Child, and Adolescent Health has been initiated to address the primary causes of maternal deaths [5]. However, India is still far from meeting the Sustainable Development Goal of reaching below 70 deaths per 100 000 live births by 2030 [6].

Any pregnancy that involves increased health risk or complications for the mother, foetus, or both during pregnancy or childbirth is referred to as high-risk pregnancy. Nearly 15% of women develop life-threatening complications during pregnancy [7], and 50-60% of women with maternal complications undergo caesarean delivery [8]. A study found that breech presentation (1.7%), heavy bleeding (7.2%), obstructed labour (7.7%), and prolonged labour (8.2%) were the primary obstetric complications in Indian women [9]. Moreover, high-risk pregnancies are responsible for 75% of perinatal deaths in India [10].

High rates of morbidity and mortality due to pregnancy-related complications are prevented through early detection, regular follow-up, and providing quality of antenatal, intranatal and postnatal interventions. To ensure comprehensive quality of obstetrics care for every pregnant woman, the Government of India launched a programme called “Pradhan Mantri Surakshit Matritva Abhiyan (PMSMA) (Prime Minister’s Safe Motherhood Campaign)” in 2016. This programme aimed to track high-risk pregnancies for counselling, management of birth preparation, and referral up until the birth outcome [11]. Though, the exact prevalence of HRP among Indian women is not known, a few studies have reported a prevalence ranging from 18.3 to 59% in different Indian communities [9,12-14]. Hence, this study was conducted to determine the prevalence and associated factors with HRP among Indian women.

## METHODS

### Study population

This study used the nationally representative cross-sectional household survey data of the India National Family Health Survey-5. This survey used probability proportionate sampling in all states and Union Territories (UTs) of India during 2019-2021. The methods, sampling and data collection procedures and tools used in the survey were reported in the NFHS-5 report [15]. We accessed the unit-level data from the Demographic Health Surreys (DHS) program (www.dhsprogram.com/data). From the individual records, we extracted the data of 28 408 currently pregnant women who were in the age group of 15-49 years. Among them, 11 625 were pregnant women who were first-time pregnant or had not given birth, while 16 783 were pregnant women who had given births.

### Outcome measures

The primary outcome of the study was high-risk pregnancies, i.e., pregnant women who had either one or more high-risk factors and were classified based on the PMSMA guidelines [16]. Using the data from the NFHS-5 survey, we identified a subset of variables for which information was available and was considered them as high-risk group. The factors are as follows:

a) Maternal risks: adolescent women whose ages range from 15 to 17 years; older women who were more than 35 years; women who were below 140 cm (cm) in height; women with a higher body mass index (BMI)≥30.0 kgmes per square metres (kg)/(m^2^) were categorised into HRP (BMI was taken into account regardless of gestational weight gain, however, the recommended cut-off of BMI for obese Asian Indian women was ≥25.0 kg/m^2^ [17]. Additionally, the gestational weight gains up to 7-11 kg for overweight women and 5-9 kg for obese women were taken into consideration for BMI≥30 kg/m^2^ in the high-risk group [18]).

b) Lifestyle risks: women who regularly smoke, use tobacco products besides cigarettes, and consume alcohol were categorised as high-risk.

c) Medical risk: women had severely anaemic (haemoglobin (Hb) of <7.0 g per decilitre (g)/(dl)).

d) Current health risks: women with their current pregnancies had any comorbidities such as diabetes, hypertension, chronic respiratory diseases including asthma, thyroid disorders, heart diseases, cancer, and chronic kidney disorders.

e) Previous birth outcome risks: pregnant women with higher birth order (five and above); Women with short birth spacing (inter-pregnancy interval i.e. last birth to time of current conception was less than 18 months) [19]; and long birth interval (more than 59 months); Women who had a history of preterm deliveries, i.e., births given at eight or fewer months (approximately <37 weeks of gestation); Women with a history of adverse birth outcomes such as miscarriage, abortion, or stillbirth; and women whose most recent delivery was a caesarean section and were classified into the HRP group.

From the above measures, there were 4555 missing data of pregnant women that were excluded from the study. Finally, 23 853 currently pregnant women were included for analyses. Among them, no high-risk factors were observed in 12 183 (50.6%) of pregnant women and categorised low-risk pregnancies (LRP). Moreover, there were 11 670 (49.4%) of pregnant women who had one or more of the above-mentioned high-risk factor(s) and categorised high-risk pregnancies.

### Explanatory variables

The independent variables included in the study were place of residence (rural, urban); educational level (no education, primary, secondary, higher); wealth index (poorest, poorer, middle, richer, richest); religion (Hindu, Muslim, Christian, others); caste (social group) (scheduled castes (SC), scheduled tribes (ST), other backward classes (OBC), and other castes (higher castes)); Indian geographical regions as used in the NFHS-5 were included as North, Northeast, East, West, Central and South [15].

### Statistical analysis

Data were extracted and statistical analysis was performed using SPSS v26 (IBM SPSS, Bengaluru, India). Univariate analysis was performed for background characteristics and expressed as percent (%). The frequency distribution of categorical variables was compared using Chi-square (χ^2^) test. Binary logistic regression analysis was carried out to examine the association between HRP with sociodemographic characteristics of the pregnant women and is reported with adjusted odds ratio (AOR) and 95% confidence interval (95% CI). Pregnancy risk was categorised as a dichotomous outcome variable and coded as “1” for high-risk and “0” for low-risk. For categorical background characteristics, the choice-based reference group was made by theoretical deliberations i.e the reference categories for place of residence, education, wealth quintile, religion, caste, and region were urban, higher education, richest, other religions, higher castes, and southern states, respectively. The statistical significance was tested at *P*-value less than 0.05.

## RESULTS

### Background characteristics of pregnant women

The contributing factors for high-risk and low-risk pregnancies among currently pregnant Indian women are shown in **Table 1**. A higher frequency of women with short birth spacing was observed in 31.1%, followed by 19.5% of women with a history of adverse birth outcomes either miscarriage, abortion, or stillbirth. Women who reported recent deliveries with caesarean sections were found in 16.4% of the last birth outcomes. Other high-risk factors were women having longer spacing (15.8%), history of preterm delivery (14.1%) and comorbidities (6.4%). Pregnancy risks within the demographic characteristics such as being adolescents, having advanced maternal age, having short stature, and having a higher BMI (≥30.0 kg/m^2^) were accounted for 1.9%, 2.2%, 2.0% and 4.7%, respectively. About 2.8% of Indian women reported with having habits of smoking or tobacco use, or drinking alcohol during their current pregnancy.

**Table 1:** Different risk factors of low-risk & high-risk pregnancies among Indian women

Currently pregnant Indian women (N = 28 408)						
**Risk factors (n)**	**Low-risk**		**High-risk**		**High-risk**	
	**Factors**	Percent*	**Factors**	Percent*	**Leading State/UT**	Percent*
**Maternal risk**						
Age, years (n = 28 408)	Adults (18-35)	95.8	Adolescents (15-17)	1.9	Tripura	10.3
			Older women (36-49)	2.2	Ladakh	14.3
Height, cm (n = 27 652)	Height ≥140	98.0	Short stature <140	2.0	Puducherry	4.8
BMI, kg/m^2^ (n = 27 618)	<30.0	95.3	Higher ≥30.0	4.7	Goa	17.4
**Lifestyle risk (n = 28 408)**	No	97.2	Smoking/tobacco/alcohol	2.8	Mizoram	43.5
Smoking	No	99.9	Yes	0.03	Mizoram	4.3
Tobacco	No	97.4	Yes	2.6	Mizoram	43.5
Alcohol	No	99.6	Yes	0.4	Arunachal Pradesh	12.5
**Medical risk**						
Anaemia (n = 27 317)	Moderate	26.3	Severe	1.4	Ladakh	16.7
	Mild	24.4				
	Not-anaemic	47.8				
**Current health risk** (n = 28 408)	No	93.6	Comorbidities	6.4	Ladakh	28.6
Diabetes	No	99.2	Yes	0.8		
Hypertension	No	97.1	Yes	2.9		
Chronic respiratory disease (asthma)	No	99.2	Yes	0.8		
Thyroid disorder	No	97.7	Yes	2.3		
Heart disease	No	99.7	Yes	0.3		
Cancer	No	99.9	Yes	0.1		
Chronic kidney disorder	No	99.8	Yes	0.2		
**Birth outcomes risk**						
Birth order (n = 28 408)	1 to 4	55.8	5 to 12	1.9	Meghalaya	10.7
	Nulliparous	42.3				
IPI (birth to conception) (months) (n = 16 754)	Space between (18-59)	53.1	Shorter spacing (<18)	31.1	Andhra Pradesh	48.1
	Nulliparous (n = 11 620)	†	Longer spacing (60-254)	15.8	AN, LA, SK	50.0
Mode of last delivery (n = 13 283)	Vaginal delivery	83.6	Caesarean delivery	16.4	Puducherry & LA	50.0
	Nulliparous (n = 11625)	†				
Last birth outcomes (n = 17 847)	Live birth	80.5	Miscarriage	14.0	Himachal Pradesh	23.3
	Primigravida (n = 10 561)	†	Abortion	3.6	Chandigarh	10.0
			Stillbirth	1.9	Sikkim	25.0
Gestational age at last birth (months) (n = 14 239)	Term (9-10)	85.9	Preterm (4-8)	14.1	Chandigarh	37.5
	Nulliparous (n = 11 625)	†				
Overall pregnancy risk‡ (n = 23 853)	LRP (n = 12 183)	50.6	HRP (n = 11 670)	49.4	Meghalaya	67.8
			Single high-risk	33.0	Tripura	41.1
			Multiple high-risk	16.4	Meghalaya	33.3

UT – Union Territory, cm – centimetres, BMI – body mass index, kg – kilogrammes, m^2^ – square metres, LRP – low-risk pregnancy, HRP – high risk pregnancy, IPI – inter-pregnancy interval, AN – Andaman Nicobar Islands, LA – Ladakh, SK – Sikkim

*The proportions are weighted to adjust for differences in probability of selection and complex survey design.

†Data were considered for pregnancy risk analysis.

‡Missing data (n = 4555) were excluded.

The frequency of leading high-risk factors among the Indian states and UTs is presented in Table S1 in the **Online Supplementary Document****.** The maternal risks such as adolescent pregnancies in Tripura (10.3%), advanced maternal age (>35 years) in Ladakh (14.3%), short stature (height below 140 cm) in Puducherry (4.8%) and higher BMI in Goa (17.4%) were observed. Lifestyle risk was higher in Northeastern states such as Mizoram in both cigarette smoking (4.3%) and tobacco chewing (43.5%) and Arunachal Pradesh in drinking alcohol (12.5%). Severe anaemia and comorbidities were higher in Ladakh i.e., 16.7% and 28.6%, respectively. The birth outcome risks such as higher birth order in Meghalaya (10.7%), short birth spacing in Andhra Pradesh (48.1%), caesarean delivery in Ladakh and Puducherry (50% each), and both adverse birth outcomes (40%) and preterm birth (37.5%) in Chandigarh were found to be higher among all states and UTs.

### Prevalence of high-risk pregnancies

**Figure 1:**
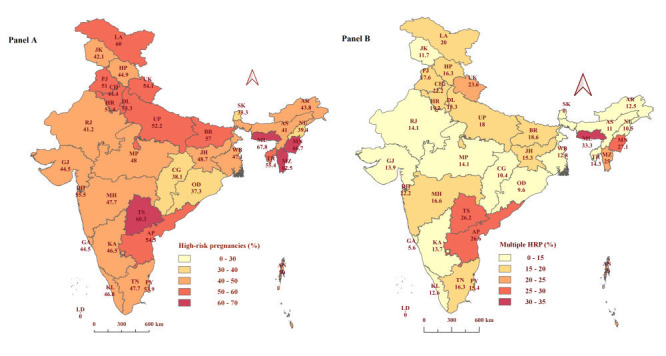
Prevalence of high-risk and multiple high-risk pregnancies among the Indian states and Union Territories. **Panel A.** Prevalence of high-risk pregnancies. **Panel B.** Prevalence of multiple high-risk pregnancies. States: Andhra Pradesh (AP), Arunachal Pradesh (AR), Assam (AS), Bihar (BR), Chhattisgarh (CG), Goa (GA), Gujarat (GJ), Haryana (HR), Himachal Pradesh (HP), Jharkhand (JH), Karnataka (KA), Kerala (KL), Madhya Pradesh (MP), Maharashtra (MH), Manipur (MN), Meghalaya (ML), Mizoram (MZ), Nagaland (NL), Odisha (OD), Punjab (PB), Rajasthan (RJ), Sikkim (SK), Tamil Nadu (TN), Telangana (TS), Tripura (TR), Uttar Pradesh (UP), Uttarakhand (UK), and West Bengal (WB). Union Territories: Andaman & Nicobar Islands (AN), Chandigarh (CH), Dadra & Nagar Haveli and Daman & Diu (DD), National Capital Territory of Delhi (DL), Jammu & Kashmir (JK), Ladakh (LA), Lakshadweep (LD), and Puducherry (PY). HRP – High-risk pregnancy

The overall prevalence of high-risk pregnancies in India was 49.4%, while 33% of women had a single high-risk and 16.4% of women had multiple high-risk factors (**Table 1**). The prevalence of high-risk and multiple high-risk pregnancies among the Indian states and UTs is shown in **Figure 1**. Northeastern states such as Meghalaya (67.8%), Manipur (66.7%) and Mizoram (62.5%) were the highest prevalence of HRPs. Lower prevalence of HRP were observed in Sikkim (33.3%), Odisha (37.3%) and Chhattisgarh (38.1%). Moreover, the highest frequency of women having multiple high-risks was recorded in Meghalaya with 33.3% (Table S2 in the **Online Supplementary Document**).

According to the sociodemographic and clinical characteristics, HRP was higher in rural women (50%), women with no formal education (61.4%) and those in the poorest wealth quintile (56.1%). Among the social groups, women belong to Christian religion (52%), and SC group (53%) had high prevalence of HRP as compared to other religion and other social groups respectively. Women from the eastern region (51.7%) had higher HRP proportion than other regions of India. Further, pregnant women in the third trimester were more with high-risk (51%) than the first (48.8%) and second trimester (48.6%). The proportion of multiple high risks were higher among women with no educational category (22.5%) than in comparison to other educated categories (**Table 2**).

**Table 2:** Socio-demographic and clinical characteristics of low-risk and high-risk pregnancies among Indian women

Demographic characteristics	Pregnancy risk (%)*				
	**n = 23 853**	**Low-risk**	**High-risk**		***P*-value**
			**Single high-risk**	**Multiple high-risks**	
**Type of residence**					
Urban	4436	52.2	31.4	16.4	<0.01
Rural	19 417	50.0	33.5	16.4	
**Education**					
No education	3817	38.6	38.9	22.5	<0.01
Primary	2551	45.0	34.4	20.7	
Secondary	9624	51.2	33.3	15.5	
Higher	7861	57.1	29.5	13.3	
**Wealth index combined**					
Poorest	5959	43.9	37.5	18.6	<0.01
Poorer	5628	49.7	33.5	16.8	
Middle	4801	51.2	32.8	16.0	
Richer	4258	54.7	30.9	14.4	
Richest	3207	55.8	28.5	15.8	
**Religion**					
Hindu	17 343	51.0	33.0	16.0	0.02
Muslim	3579	49.0	32.7	18.3	
Christian	2031	48.0	34.3	17.7	
Others	900	49.8	33.2	17.0	
**Castes (social group) (n = 21 886)**					
Scheduled castes	4918	47.0	34.7	18.3	<0.01
Scheduled tribes	4929	53.0	32.0	15.0	
Other backward classes	9070	51.8	32.6	15.7	
Others castes	3775	49.8	33.6	16.6	
**Region**					
North	4561	54.2	30.0	15.8	<0.01
North-East	3585	53.5	31.9	14.6	
East	4654	48.3	35.8	16.0	
West	2069	53.5	31.0	15.6	
Central	6040	50.0	33.5	16.5	
South	2944	49.7	31.7	18.6	
**Trimesters**					
First	7089	51.2	32.7	16.1	<0.01
Second	9217	51.4	33.0	15.7	
Third	7547	49.0	33.4	17.6	

*The proportions are weighted to adjust for differences in probability of selection and complex survey design.

### Determinants of high-risk pregnancies

The factors associated with high-risk pregnancies is presented in **Table 3**. Women who had no formal education were two-times having higher odds of high-risk(s) (AOR = 2.02; 95% CI = 1.84-2.2) than women with higher education. Further, women under poorest wealth quintile were 33% with more high-risk than the richest category (AOR = 1.33; 95% CI = 1.18-1.49). Women under ST group had lower odds of HRP than in comparison to higher castes category (AOR = 0.69; 95% CI = 0.62-0.78). Northern states women were less likely to have high-risk than those in southern states (AOR = 0.72; 95% CI = 0.65-0.79).

**Table 3:** Determining factors associated with high-risk pregnancies among Indian women

Characteristics	HRP (n = 22 692)
	**AOR (95% CI)**
**Type of residence**	
Rural	0.93 (0.87-1.00)
Urban	Reference
**Education**	
No education	2.02 (1.84-2.22)*
Primary	1.62 (1.46-1.79)*
Secondary	1.25 (1.16-1.33)*
Higher	Reference
**Wealth index combined**	
Poorest	1.33 (1.18-1.49)*
Poorer	1.15 (1.04-1.29)†
Middle	1.12 (1.01-1.24)†
Richer	1.00 (0.91-1.10)
Richest	Reference
**Religion**	
Hindu	0.80 (0.66-0.98)†
Muslim	0.83 (0.67-1.02)
Christian	0.98 (0.75-1.29)
Others	Reference
**Caste (social group)**	
Scheduled castes	0.96 (0.88-1.05)
Scheduled tribes	0.69 (0.62-0.78)*
Other backward classes	0.83 (0.77-0.90)*
Others castes	Reference
**Region**	
North	0.72 (0.65-0.79)*
North-East	0.77 (0.64-0.93)*
East	0.78 (0.71-0.85)*
West	0.81 (0.73-0.90)*
Central	0.82 (0.75-0.89)*
South	Reference

HRP – high-risk pregnancy, AOR – adjusted odds ratio, CI – confidence interval

*P < 0.001.

†*P* < 0.05.

## DISCUSSION

According to our study, the country’s overall prevalence of HRP was 49.4%, which is higher than that of adjacent countries like Nepal (14.4%) [20], Bangladesh (41.5%) [21], China (24.5%) [22], as well as other low-middle income countries like Ethiopia (26.4%) [23] and the Democratic Republic of Congo (33.1%) [24]. Among all pregnancies, nearly one-third of Indian pregnant women had at least one high-risk factor and sixteen percent of women had multiple-high risks. These proportions are comparatively lower than in single high-risk and multiple high-risks reported in the Korean population [25]. High-risk factors among pregnant women were more prevalent in northeastern states such as Meghalaya, Manipur, Mizoram, and a southern state of Telangana. The prevalence of multiple high-risk pregnancies was also higher in Meghalaya, Manipur, Andhra Pradesh and Telangana states. In those states, risk factors such as advanced maternal age, smoking and tobacco use, alcohol consumption, higher birth orders and short birth spaces were found to be more common. On the other hand, the lowest prevalence was observed in Sikkim, Odisha, and Chhattisgarh states. However, these findings revealed that states with higher Infant Mortality Rates (IMR)/MMR had lower prevalence of high-risk factors [26]. Hence, this study emphasises that greater attention is required to provide quality maternal and obstetric care during pregnancy and delivery.

The short birth spacing was the primary factor contributing to the high prevalence of high-risk pregnancies across the country. The major problem of short-birth spacing was that half of the Indian women were not using contraception to delay their next pregnancy [27]. According to NFHS-5 survey, infant and child mortality rates were higher in high-risk births delivered by Indian mothers who have multiple risk factors such as being too young or very old at birth, having higher birth orders, and having birth intervals that are too short [28]. The under five-mortality rate for shorter birth intervals was reported to be twice as high as the rate for birth intervals of three or more years [28]. A shorter inter-pregnancy interval was closely associated with premature rapture of membranes, abruptio placenta, placenta praevia, and uterine rupture in women with previous caesarean deliveries [29]. Hence, additional research is needed to address the issue of short birth spacing among Indian women.

Caesarean deliveries, preterm deliveries, and adverse birth outcomes such as miscarriage, abortion, and stillbirth were other key contributing factors of HRP, these findings are aligned with other research studies [9,30]. There were substantial variations in obstetric care approaches among the pregnant women due to low socioeconomic status, being less educated, and belonging to scheduled castes or scheduled tribes, which contributed to variations in newborn outcomes [31]. Notably, our earlier study found an increased prevalence of miscarriage and stillbirth among Indian women [32]. A systematic review showed that women who had previous caesarean deliveries within a shorter duration from the last birth were at increased risk of uterine rupture, blood transfusions, and maternal morbidity [33]. Bad obstetric history, pregnancy-induced hypertension, anaemia, and poor maternal nutrition were the other main factors associated with high-risk, which led to low birth weight and infant malnutrition [30,34]. In addition, factors like maternal age, less weeks of gestation, short-birth spacing, and higher birth orders were the other high-risk factors, which are interplaying factors with the weight of the newborn [34]. Recently, Kruk and her group found that the likelihood of neonatal mortality and stillbirth was higher among high-risk pregnant Indian women [31]. Hence, these pregnant women are required to receive prompt and effective interventions during pregnancy and delivery in order to decrease maternal and neonatal morbidities and mortality.

Our study found that over four percent of women had a higher BMI, and more than six percent of women reported having comorbidities during their current pregnancies. Obesity is associated with a greater risk of developing gestational diabetes, cardiovascular disease, venous thromboembolism, and infections, particularly in older women, and these mothers have a higher risk of perinatal fatalities [35]. Increased BMI and excessive gestational weight gain during pregnancy were found to be associated with infant’s birth weight [36,37], also developing preeclampsia in women [38], and requiring more birth interventions like caesarean delivery [39]. Comorbidities, or pregnancy-related complications, were attributed to the greatest risk of maternal deaths. Empowered Action Group (EAG) States in India such as Assam, Bihar, Chhattisgarh, Jharkhand, Madhya Pradesh, Odisha, Rajasthan, Uttar Pradesh and Uttarakhand were reported to have higher rates of maternal deaths, and complications during pregnancy were also higher in these states [40]. In our study, adolescent pregnancy, lifestyle factors, higher birth order, birth spacing issues, and preterm delivery were more frequently reported in the EAG states. Several non-EAG states have high HRP rates, but due to the continuum of care and excellent quality treatment, the maternal mortality rate is lower than in those with lower prevalence of HRP states. This suggests that by improving the quality of obstetric care and medical health facilities in the EAG states lead to way forward in reduction of IMR and MMR. More studies are required in these directions to address these issues among Indian women.

Women under the lowest wealth quintiles and no education were the independent predictive factors of HRP. These results were corroborated with other research in a similar manner [41]. Women who were from the Christianity had a greater risk of high-risk than women of other religions. While ST women had a lower likelihood of high-risk and multiple high-risk than the higher-caste women, Women in southern states were more likely to experience high-risk and multiple-high-risk than those in northern states, and it could be due to the higher frequency of women with obesity, comorbidities, short birth spacing and more in caesarean section delivery [42].

Effective primary care referral systems will aid in the early detection of high-risk conditions, which will help to provide early management and quality obstetric care for complicated pregnancies. A multidisciplinary team of obstetricians, maternal-foetal medicine specialists, cardiologists, nephrologists, diabetologists and psychologists are necessary for providing care to high-risk pregnant women, particularly those who have illnesses including diabetes, hypertension, heart disease and mental health disorders. Every pregnant woman should utilise the government's health facilities and maternal health care services, which include a comprehensive ANC package, prenatal counselling, new-born care, breastfeeding support and emergency obstetric and newborn care facilities. Further, upon empowering the Accredited Social Health Activist (ASHA) workers who will assist and counsel the couples for delaying the period from one birth to another and they can also do regular follow-up of high-risk pregnant women for safe delivery at referral health care facilities.

The strength of our study includes diversity of data set, a larger sample size, and the fact that such analysis is not available at the national level. We identified some of the sociodemographic and geographic groups where the population of women with high-risk pregnancy is highly concentrated, which is important from the policy and programme perspectives. However, our study has some limitations. This study does not include pregnancy-related complications such as gestational diabetes mellitus, multiple gestations, and infections during the current pregnancy. The frequency data set of mode of delivery and gestational age at delivery for women having a longer duration of birth spacing were not available; those could add more value to our study. Another limitation is the fact that reporting of current health issues such as hypertension, respiratory disease, etc. was self-reported and undiagnosed. It is also possible that some of the smaller states/UTs with higher prevalence are probably due to a smaller sample size.

## CONCLUSIONS

In India, the high prevalence of high-risk pregnancies is a matter of concern, and it could be a probable factor for high maternal and neonatal morbidity and mortality in the country. The Northeastern states such as Meghalaya, Manipur and Mizoram exhibit the highest frequency of high-risk pregnancies. Further investigations are required focusing on factors such as advanced maternal age, tobacco use, pre-existing diseases, and adverse birth outcome issues. Short birth spacing contributes to the major share to HRP prevalence in India. Appropriate measures on birth spacing issues through policy and programmes and creating public awareness and education of women in the long run will help for further improvement of maternal and neonatal outcomes. The government should focus on regional-specific health issues and regional policies on socio-behavioural interventions to improve lifestyle practices, particularly in the Northeast. States with a lower prevalence of HRP witnessed higher maternal deaths in Odisha, Chhattisgarh and Assam, suggesting that need of more clinical and laboratory investigations could be the best possible way to address the health issues and also to improve the maternal health and outcomes. There is also a need of regular monitoring the high-risk pregnancies through health system, data harmonisation and tracking to prevent adverse maternal and neonatal outcomes.

## Additional material


Online Supplementary Document
