# Supplementary material for: High-risk pregnancy in India: Prevalence and contributing risk factors – a national survey-based analysis
Source: J Glob Health. 2023 Sep 15;13:04116. doi: 10.7189/jogh.13.04116 (PMC10502764; doi:10.7189/jogh.13.04116)
Supplement: Online Supplementary Document [file jogh-13-04116-s001.pdf]

**Supplementary Table 1:** Stratification of high-risk factors among pregnant women with top five Indian States and Union Territories

| S. No. | High-risk factors (N=28408 pregnant women) | Top five States/UTs | |
|---|---|---|---|
| | | **States/UTs** | **Percent** |
| **a)** | **Maternal age risks** | | |
| i) | Adolescence pregnancy (15-17 years) | Tripura | 10.3 |
| | | West Bengal | 7.0 |
| | | Bihar | 3.2 |
| | | Maharashtra | 2.8 |
| | | Andhra Pradesh | 2.8 |
| ii) | Advanced maternal age (36-49 years) | Ladakh | 14.3 |
| | | Mizoram | 13.6 |
| | | Meghalaya | 13.3 |
| | | Goa | 9.1 |
| | | Manipur | 8.5 |
| iii) | Height short stature (< 140 cm) | Puducherry | 4.8 |
| | | Bihar | 4.1 |
| | | Tripura | 3.9 |
| | | Meghalaya | 2.7 |
| | | Uttar Pradesh | 2.6 |
| iv) | Body mass index $\geq 30$ kg/m$^2$ | Goa | 17.4 |
| | | Sikkim | 16.7 |
| | | Andhra Pradesh | 13.1 |
| | | Tamil Nadu | 12.0 |
| | | Dadra & Nagar Haveli and Daman & Diu | 11.1 |
| **b)** | **Lifestyle Factors** | | |
| i) | Smoking (cigarettes) | Mizoram | 4.3 |
| | | Jammu & Kashmir | 0.3 |
| | | Rajasthan | 0.1 |
| | | West Bengal | 0.1 |
| | | Jharkhand | 0.1 |
| ii) | Tobacco (chewing) | Mizoram | 43.5 |
| | | Manipur | 32.2 |
| | | Meghalaya | 31.3 |
| | | Tripura | 19.2 |
| | | Nagaland | 14.3 |
| iii) | Alcohol (drinking) | Arunachal Pradesh | 12.5 |
| | | Assam | 4.1 |
| | | Chhattisgarh | 2.4 |
| | | Jharkhand | 1.8 |
| | | Telangana | 1.7 |
| **c)** | **Medical risk** | | |
| i) | Severe anemia (Hb <7.0 g/dl) | Ladakh | 16.7 |
| | | Puducherry | 4.8 |
| | | Uttarakhand | 3.1 |
| | | Meghalaya | 2.7 |
| | | Haryana | 2.2 |
| **d)** | **Pre-existing diseases*** | Ladakh | 28.6 |
| | | Sikkim | 16.7 |
| | | Meghalaya | 15.2 |
| | | Kerala | 15.0 |
| | | Telangana | 14.2 |
| **e)** | **Birth outcome risk** | | |
| i) | High birth order (5 & above) | Meghalaya | 10.7 |
| | | Mizoram | 4.3 |
| | | Bihar | 4.0 |
| | | Uttarakhand | 3.8 |

| | | | |
|---|---|---|---|
| | | Uttar Pradesh | 3.2 |
| ii) | Short birth space (<18 months) | Andhra Pradesh | 48.1 |
| | | Bihar | 43.2 |
| | | Telangana | 40.6 |
| | | Dadra & Nagar Haveli and Daman & Diu | 40.0 |
| | | Uttar Pradesh | 36.8 |
| iii) | Long birth space (≥60 months) | Sikkim | 50.0 |
| | | Ladakh | 50.0 |
| | | Andaman & Nicobar Islands | 50.0 |
| | | Tripura | 47.4 |
| | | Puducherry | 46.7 |
| iv) | Caesarean delivery | Ladakh | 50.0 |
| | | Puducherry | 50.0 |
| | | Telangana | 49.1 |
| | | Jammu & Kashmir | 44.4 |
| | | Tamil Nadu | 38.0 |
| v) | Adverse birth outcomes# | Chandigarh | 40.0 |
| | | NCT of Delhi | 27.0 |
| | | Himachal Pradesh | 26.6 |
| | | Manipur | 26.2 |
| | | Haryana | 25.9 |
| vi) | Preterm birth (gestation ≤8 months)$ | Chandigarh | 37.5 |
| | | Uttarakhand | 31.5 |
| | | Himachal Pradesh | 27.9 |
| | | Dadra & Nagar Haveli and Daman & Diu | 20.0 |
| | | Arunachal Pradesh | 20.0 |

UTs- Union Territories; Hb-Haemoglobin; Nct- National Capital Territory.
* Health risk including diabetes, hypertension, chronic respiratory disease, thyroid disorder, heart disease, cancer, chronic kidney disease).
# Adverse birth outcomes including miscarriage, abortion & stillbirth
$ Women whose last delivery with 8 or less months of gestational period was considered.
The proportions are weighted to adjust for differences in probability of selection and complex survey design.

**Supplementary Table 2:** Prevalence of low-risk and high-risk pregnancies among the Indian States and Union Territories.

| Regions | States / Union Territories | Pregnancy risk (%) | | | |
|---|---|---|---|---|---|
| | | Low-risk (n=12183) | High-risk pregnancy (HRP) (n=11670) | | |
| | | | Single HRP | Multiple HRP | Total HRP |
| North | Chandigarh (CH) | 55.6 | 22.2 | 22.2 | 44.4 |
| | Delhi (DL) | 46.7 | 38.0 | 15.3 | 53.3 |
| | Haryana (HR) | 48.6 | 32.2 | 19.2 | 51.4 |
| | Himachal Pradesh (HP) | 55.1 | 28.6 | 16.3 | 44.9 |
| | Jammu & Kashmir (JK) | 57.9 | 30.4 | 11.7 | 42.1 |
| | Ladakh (LA) | 40.0 | 40.0 | 20.0 | 60.0 |
| | Punjab (PB) | 49.0 | 33.4 | 17.6 | 51.0 |
| | Rajasthan (RJ) | 58.8 | 27.1 | 14.1 | 41.2 |
| | Uttarakhand (UK) | 45.9 | 30.5 | 23.6 | 54.1 |
| North-East | Arunachal Pradesh (AR) | 56.2 | 31.3 | 12.5 | 43.8 |
| | Assam (AS) | 59.0 | 30.0 | 11.0 | 41.0 |
| | Manipur (MN) | 33.3 | 39.6 | 27.1 | 66.7 |
| | Meghalaya (ML) | 32.2 | 34.5 | 33.3 | 67.8 |
| | Mizoram (MZ) | 37.5 | 37.5 | 25.0 | 62.5 |
| | Nagaland (NL) | 60.6 | 28.9 | 10.5 | 39.4 |
| | Sikkim (SK) | 66.7 | 33.3 | 0.0 | 33.3 |
| | Tripura (TR) | 44.6 | 41.1 | 14.3 | 55.4 |
| East | Bihar (BR) | 43.0 | 38.4 | 18.6 | 57.0 |
| | Jharkhand (JH) | 51.3 | 33.4 | 15.3 | 48.7 |
| | Odisha (OD) | 62.7 | 27.7 | 9.6 | 37.3 |
| | West Bengal (WB) | 52.9 | 34.3 | 12.8 | 47.1 |
| West | Dadra & Nagar Haveli and Daman & Diu (DD) | 44.5 | 33.3 | 22.2 | 55.5 |
| | Goa (GA) | 55.5 | 38.9 | 5.6 | 44.5 |
| | Gujarat (GJ) | 55.5 | 30.6 | 13.9 | 44.5 |
| | Maharashtra (MH) | 52.3 | 31.1 | 16.6 | 47.7 |
| Central | Chhattisgarh (CG) | 61.9 | 27.7 | 10.4 | 38.1 |
| | Madhya Pradesh (MP) | 52.0 | 33.9 | 14.1 | 48.0 |
| | Uttar Pradesh (UP) | 47.8 | 34.2 | 18.0 | 52.2 |
| South | Andaman & Nicobar Islands (AN) | 50.0 | 25.0 | 25.0 | 50.0 |
| | Andhra Pradesh (AP) | 45.5 | 27.9 | 26.6 | 54.5 |
| | Karnataka (KA) | 53.5 | 32.8 | 13.7 | 46.5 |
| | Kerala (KL) | 53.2 | 34.2 | 12.6 | 46.8 |
| | Lakshadweep LD) | 100.0 | 0.0 | 0.0 | 0.0 |
| | Puducherry (PY) | 46.1 | 38.5 | 15.4 | 53.9 |
| | Tamil Nadu (TN) | 52.3 | 31.4 | 16.3 | 47.7 |
| | Telangana (TS) | 39.7 | 34.1 | 26.2 | 60.3 |
